# Determination of Genetic Diversity in *Chilo partellus*, *Busseola fusca*, and *Spodoptera frugiperda* Infesting Sugarcane in Southern Malawi Using DNA Barcodes

**DOI:** 10.3390/insects9030074

**Published:** 2018-06-22

**Authors:** Trust Kasambala Donga, Richard Meadow

**Affiliations:** 1 Norwegian University of Life Sciences, P.O. Box 5003 NMBU, Ås NO-1432, Norway; richard.meadow@nmbu.no; 2 Lilongwe University of Agriculture and Natural Resources, P.O. Box 219 Lilongwe, Malawi

**Keywords:** Sugarcane, Lepidoptera, Noctuidae, Crambidae, population genetics, COI gene

## Abstract

Sugarcane is one of the most valuable crops in the world. Native and exotic Lepidopteran stemborers significantly limit sugarcane production. However, the identity and genetic diversity of stemborers infesting sugarcane in Malawi is unknown. The main objectives for this study were to identify and determine genetic diversity in stemborers infesting sugarcane in Malawi. We conducted field surveys between June 2016 and March 2017 in the Lower Shire Valley district of Chikwawa and Nsanje, southern Malawi. Molecular identification was based amplification the partial cytochrome oxidase subunit I (COI) gene region. Phylogenetic trees for sequences were generated and published GenBank accessions for each species were constructed. We found that Malawi *Busseola fusca* (Lepidoptera: Noctuidae) specimens belonged to clade II, *Spodoptera frugiperda* sp. 1 (Lepidoptera: Noctuidae) and *Chilo partellus* (Lepidoptera: Crambidae) were infesting sugarcane. Interspecific divergence ranged from 8.7% to 15.3%. Intraspecific divergence was highest for *B. fusca*, 3.6%. There were eight haplotypes for *B. fusca*, three for *S. frugiperda* and three for *C. partellus*. The importance of accurate species identification and genetic diversity on stemborer management is presented.

## 1. Introduction

Sugarcane is an important cash crop throughout the tropics. Southern Africa has the lowest yields of sugarcane (hg/ha), 82% less than the world average [1,2]. For over 50 years, sugarcane has been grown for processing purposes in Malawi. Production is intense, year-round, and under irrigation in estates. Smallholder farmers contribute 20% to the national production [3,4]. Some of these farmers grow sugarcane under irrigation while others solely depend on rainfall. Some farmers grow the crop either as an intercrop or as a monocrop or border crop. The crop is row intercropped with maize (*Zea mays* L.), sorghum (*Sorghum bicolor* L. Moench), vegetables, or a combination, during the dry season (May to November). Due to continuous monocropping on the large commercial estates, pest prevalence is high. In addition, continuous pest refugia are provided by intercropping or rotating sugarcane with cereals such as maize and sorghum.

A myriad of arthropod pests infests sugarcane. About 50 species of Lepidopteran moths belonging to three families, namely Noctuidae, Crambidae, and Pyralidae, infest sugarcane [5,6]. Within the family Pyralidae, *Eldana saccharina* Walker, a native of Africa is considered a serious pest of sugarcane [6]. It is widely distributed in sub-Saharan countries [7]. The species of *Chilo* (Crambidae), namely *C. partellus* and *C. sacchariphagus*, are also economic pests of sugarcane in eastern and southern Africa [8]. *C. partellus* is an invasive pest that was introduced from India to Africa. Sugarcane is also a host for *C. orichalcociliellus* [9]. *Sesamia calamitis*, *S. creta*, and *Busseola* (Noctuidae), although considered as main pests of maize and sorghum [9,10], can also infest sugarcane. The larvae of these moths bore into and feed internally on stem tissue. The larval entry points on the stem provide entrance for fungal diseases. In younger plants, larval feeding results in death of the apical meristem, a condition called ‘dead hearts.’ In older plants, feeding damage results in increased risk of lodging. In addition, the quality and quantity of yield (sucrose) is also affected.

Multiple stemborer species may infest a field or individual plants [11,12]. However, variation exists in the pest status of these pests on sugarcane in Africa [7]. In South Africa and Zimbabwe, *E. saccharina* Walker is a major pest [13]. In Mozambique, the main stemborer species attacking sugarcane is *C. sacchariphagus* Bojer [14,15], while in Botswana it is *Chilo partellus* Swinhoe [16]. Although *E. saccharina* and *Sesamia calamistis* Hampson are present in Ethiopia, they are not economic pests on small-scale sugarcane farmers’ fields [6]. Outbreaks of the fall armyworm, *Spodoptera frugiperda* (J.E. Smith) were first reported in Africa in 2016 [16,17]. During the 2016–2017 cropping season, *S. frugiperda* was reported to infest maize in several African countries. Although *S. frugiperda* prefers maize, it can also infest sugarcane [16].

The cytochrome c oxidase subunit 1 (COI) mitochondrial DNA (mtDNA) gene is widely used in identification and determination of insect population structure [18,19]. Genetic diversity in *B. fusca* populations is well documented. *B. fusca* populations cluster into three clades namely West Africa (W), Kenya I (KI), and Kenya II (KII) [20,21,22]. Clade KII comprises *B. fusca* species from eastern and central Africa [19,20]. On the contrary, studies establishing genetic differentiation in *C. partellus* in Africa are limited. A study by Sezonlin M. et al. [19] found that *C. partellus* populations collected from maize and sugarcane fields in South Africa and Swaziland were genetically similar. In that study, 11 *C. partellus* larvae from South African sugarcane were analyzed. The sequences generated in that study were not compared with sequences from other countries to determine genetic variations. Also, there are significant differences in the climate and geography of Malawi from that of South Africa. It has been suggested that gene flow between organisms of the same species might be restricted by physical barriers such as mountains and major rivers which may lead to speciation overtime [18].

Lack of knowledge of pest species identity and composition makes it difficult to properly address the problem in the context of integrated pest management. Published records indicate the occurrence of *C. partellus*, *C. orichalcociliellus*, and *B. fusca* in Malawi [23,24,25]. An unknown species of *Chilo* and *C. sacchariphagus* are reported in unpublished records of sugar estates in Chikwawa, southern Malawi. There is no record of *E. saccharina* occurrence in the country even though the pest occurs in neighbouring Mozambique [7]. Currently, stemborer management is based on varietal mixtures. Chemical control is less effective because of the cryptic nature of the pests. Biological control using the egg parasitoid *Trichogramma chilonis* is also recommended. Research on occurrence of fungal pathogens with insect control potential began in 2015. The success of such efforts hinges on correct pest identification and characterization, which is currently lacking. Our aims in this study were to accurately identify stemborer infesting sugarcane in Chikwawa and Nsanje Districts, southern Malawi using the COI gene, and determine diversity and relatedness among stemborer species with published reference sequences from GenBank. Results of this study will contribute to effective management of stemborers in the Malawi sugarcane industry.

## 2. Materials and Methods

### 2.1. Survey Sites

**Figure 1:**
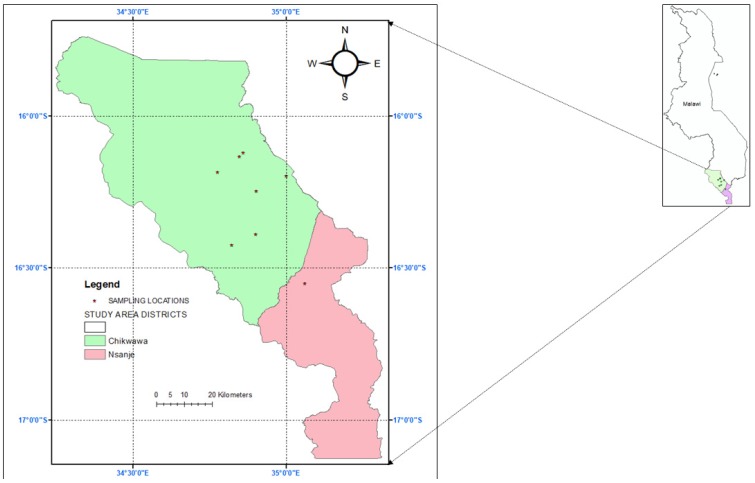
Map of localities where *Busseola fusca, Chilo partellus*, and *Spodoptera frugiperda* were sampled in Chikwawa and Nsanje districts, southern Malawi.

Sugarcane is grown in the Nkhata Bay, Nkhota Kota, Salima, Chikwawa, and Nsanje districts (Figure 1). There are several estates in Chikwawa, namely: Kasinthula, Sande, Nchalo, and Alumenda Estates. Kaombe Estate is located in Nsanje District. In addition to estates, smallholder farmers typically grow sugarcane in seasonal low-lying wetlands (locally called ‘dimba’) under rainfed conditions and residual moisture. No fertilizers or manure or pesticides are applied. The Shire River provides water for irrigation in Chikwawa and Nsanje districts, respectively.

### 2.2. Survey Methodology

Commercial sugarcane production in Malawi dates back to 1968 [26]. Surveys were conducted in 48 fields belonging to Kasinthula, Nchalo, Alumenda, Kaombe, and Sande Estates, and smallholder fields located in agricultural extension planning areas (EPA) of Mbewe, Kalambo, Livunzu, and Mikalango in Chikwawa and Nsanje districts in southern Malawi from June 2016 to March 2017. All larvae collected were stored in 70% alcohol in 30 mL sealed vials and kept at 4 °C. The vials had labels corresponding to a datasheet that had the following information: collection date, location, plant damage, life stage, and number of larvae collected. The samples were shipped to the South African Sugarcane Research Institute (SASRI), Mount Edgecombe, KwaZulu-Natal, South Africa and the Norwegian University of Life Sciences, Ås, Norway for identification and molecular characterization, respectively.

### 2.3. Morphological and Molecular Identification

Morphological identification of the collected larvae to genus or species level, or both, was based on external anatomy (chaetotaxy and crochet arrangement) based on identification keys provided by Meijirman and Ulenberg [27]. Fall armyworm samples were identified using FAO [28] descriptions of the pest. A dissecting microscope was used in examining the larval specimens. Larvae were allocated to three species namely: *Busseola fusca*, *Chilo partellus*, and *Spodoptera frugiperda*. Molecular tools described below were used to confirm species and identify unknown species.

### 2.4. DNA Extraction and Amplification

A total of 217 larvae were morphologically identified to species level, two specimens to genus level and two to order level, respectively. At least one larval specimen from each of the identified species/genera/order and from each of the 48 fields sampled were sent for DNA based identification at the South African Sugarcane Research Institute (SASRI), Mount Edgecombe, KwaZulu-Natal, South Africa. DNA was extracted from whole insects (if very small) or a body part, using the GeneJet Genomic DNA Purification kit (Thermo Scientific, Waltham, MA, USA) according to the manufacturer’s instructions. The DNA was quantified using a NanoDrop Spectrophotometer (Thermo Scientific, Waltham, MA, USA). PCR amplification was conducted using the KAPA 2G Robust PCR Kit (Kapa Biosystems, Cape Town, South Africa) with approximately 50 ng DNA template. The final reaction conditions were as follows: 1x Kapa2G Buffer A, 0.2 mM dNTP mix, 0.5 μM each HCO 2198 and LCO 1490 and 0.5 units Kapa2G Robust DNA Polymerase. The DNA primer sequences used were HCO 2198 (5′ TAAACTTCAGGGTGACCAAAAAATCA 3’) and LCO 1490 (5’ GGTCAACAAATCATAAAGATATTG 3′) [29].

PCR reactions were conducted in an Applied Biosystems Veriti Thermal Cycler (Applied Biosystems, Marina Bay, Singapore). The thermal cycling profile was 94 °C for 2 min, followed by 35 cycles of 94 °C for 30 s, 55 °C for 50 s and 72 °C for 90 s. Final extension was at 72 °C for 10 min. PCR products were purified using a DNA Clean and Concentrator kit (Zymo Research, Irvine, CA, USA) according to the manufacturer’s instructions.

### 2.5. DNA Sequencing

DNA sequencing was conducted using the BigDye Terminator v3.1 Cycle Sequencing kit (Applied Biosystems, Foster City, CA, USA) according to the manufacturer’s instructions. Sequencing reactions were conducted in an Applied Biosystems Veriti Thermal Cycler using the BigDye Terminator v3.1 kit recommended thermal cycling profile. Sequencing products were purified using the BigDye XTerminator Purification Kit (Applied Biosystems, Foster City, CA, USA) according to manufacturer’s instructions. DNA sequences were analysed by capillary electrophoresis using the ABI3500 Genetic Analyser (Applied Biosystems, Foster City, CA, USA) following standard operating protocols.

### 2.6. Sequence Analysis

DNA sequences were trimmed on the 5′ and 3′ ends to remove poor quality sequences using CLC Main workbench v7.0.1 (QIAGEN, Hilden, Germany). The putative identities for each sequence were established by comparison with the DNA barcode sequence repository of the BOLD database. Sequences were aligned using ClustalW [30] with default settings in BioEdit 7.2.5 [31]. In addition, reference sequences from GenBank were downloaded (Table 1) and incorporated in phylogenetic study. A neighbor-Joining (NJ) and maximum likelihood (ML) analysis based on K-2 parameter model [32] with complete gap deletion and resampled with 1000 bootstrap replications were done using all sequences generated in the study and the reference sequences. We used the model selection option in Mega6 [33] to find the best-fit substitution model for our dataset. Based on the lowest Bayesian Information Criterion (BIC) value, Tamura 3-parameter with discrete Gamma distribution (T92 + I) [33] fit the dataset best. Maximum Likelihood (ML) was performed in using the best-fit model and clusters and 1000 bootstrap replications were used to support clusters. Separate phylogenetic analyses with reference sequences were performed for *B. fusca* (*n* = 11) and *S. frugiperda* (*n* = 11) in Mega6. DnaSP v5 [34] was used to calculate DNA polymorphism parameters: number of polymorphic (segregating) sites, S; number of haplotypes, h; haplotype (gene) diversity, Hd; and nucleotide diversity, Pi (π). All sequences produced have been submitted to GenBank.

## 3. Results

### 3.1. Occurrence of Busseola fusca, Chilo partellus, and Spodoptera frugiperda in Sugarcane Fields

#### 3.1.1. Morphological Identification

**Figure 2:**
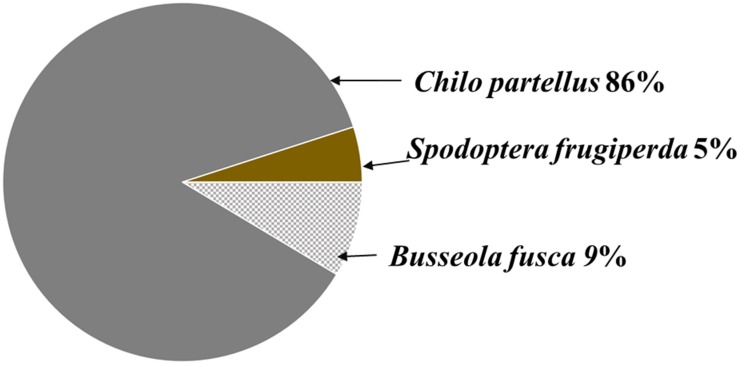
Percent distribution of *Busseola fusca*, *Chilo partellus*, and *Spodoptera frugiperda* (based on morphological) collected from sugarcane fields in Chikwawa and Nsanje districts, southern Malawi (*n* = 217).

From 48 sugarcane fields (Table S1), 221 larvae were collected. Based on morphology, we identified 219 larvae as Lepidoptera and 2 as Diptera. The 219 Lepidopteran larvae belonged to four genera namely *Chilo, Busseola, Spodoptera*, and *Sesamia.* Morphologically, *Sesamia* spp could not be identified to species level. However, we identified the remaining Lepidopteran larvae as *Busseola fusca*, *Chilo partellus*, and *Spodoptera frugiperda* (Figure 2).

#### 3.1.2. DNA Based Identification

**Figure 3:**
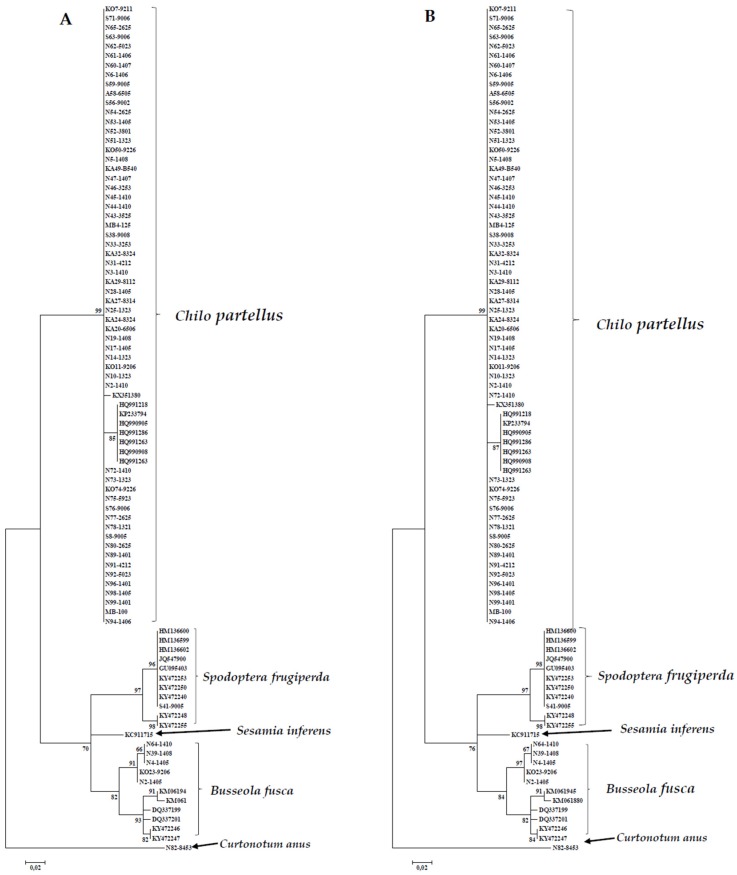
Phylogenetic tree inferred using the Maximum Likelihood (ML)) method of mtDNA CO1 region of *Busseola fusca*, *Chilo partellus*, and *Spodoptera frugiperda* sequences obtained from sugarcane fields in southern Malawi together with reference sequences from other African countries. (**A**) The tree is based on the Kimura 2-parameter method. (**B**) The tree is based on Tamura 3-parameter model with evolutionarily invariable (T92 + I). Both trees were resampled with 1000 bootstrap replicates. Bootstrap support values on the branches are given.

DNA was extracted from, amplified, and sequenced for 65 samples. Based on initial BOLD searches; 59 sequences were identified as *C. partellus*, 4 as *B. fusca*, 1 as *S. frugiperda* and *C. anus Curtonotum anus* (Curtonotidae: Diptera). Initial GenBank searches could not resolve the identity of the *Sesamia* larva as the top 20 searches showed 94.5% identity match as *S. inferens* and the same percentage to *B. fusca*. However, based on phylogenetic analyses, the sequence for this larva aligned with *B. fusca* with higher bootstrap branch support values (Figure 3).

### 3.2. Sequence Analysis

Sixty-five sequences of varying length (average 585 bp) were generated for *B. fusca*, *C. partellus*, and *S. frugiperda*. Sequences were trimmed to 539 bp and used in analyses. A total of 25 sequences were downloaded from GenBank for comparisons and comprised *B. fusca* (*n* = 7), *C. partellus* (*n* = 8) and *S. frugiperda* (*n* = 10) (Table 2). A NJ and ML tree was produced for all sequences (*n* = 90) from this study and GenBank. Both NJ and ML trees had comparable topologies with clearly differentiated clades denoting distinct species (Figure 3). The first clade included all *C. partellus* specimens and their corresponding reference sequences (Figure 3). The second clade consisted of *S. frugiperda* individuals and the third cluster had *B. fusca* samples (Figure 3).

**Figure 4:**
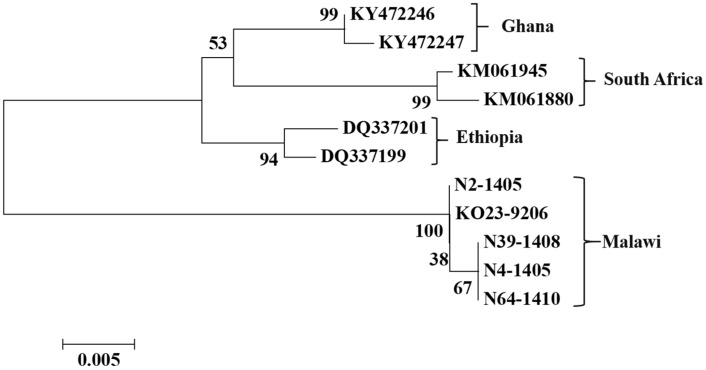
Phylogenetic tree inferred using the Neighbor-Joining (NJ) method of 11 mtDNA CO1 region of *Busseola fusca* sequences obtained from sugarcane fields in southern Malawi together with reference sequences from other African countries. The tree is based on the Kimura 2-parameter method. The tree was resampled with 1000 bootstrap replicates. Bootstrap support values on the branches are given.

**Figure 5:**
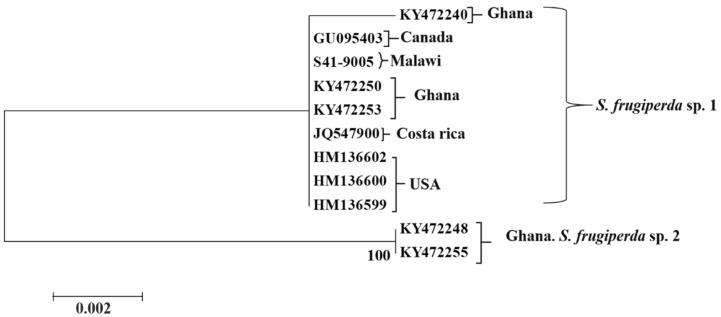
Phylogenetic tree inferred using the Neighbor-Joining (NJ) method of 11 mtDNA CO1 region of *Spodoptera frugiperda* sequences obtained from sugarcane fields in southern Malawi together with reference sequences from other African countries. The tree is based on the Kimura 2-parameter method and 1000 bootstrap duplications.

Based on both NJ and ML analyses of the alignment of the alignment with COI gene sequences, we found that all *C. partellus* clustered with the reference sequences (Figure 3). The COI gene sequenced Malawian *C. partellus* samples formed one cluster which was strongly supported (bootstrap support value, 99%). As depicted in Figure 4, *B. fusca* individuals formed four distinct clusters corresponding to country of origin. Finally, the *S. frugiperda* sequence generated in this study aligned with *S. frugiperda* sp.1 from Ghana and the Americas (Figure 5). Mean between groups genetic distances were: *S. frugiperda* and *C. partellus*, 13.5%; *C. partellus* and *B. fusca*, 15.3%; *B. fusca* and *S. frugiperda*, 8.7%. Mean within group species divergence were 0.3% for *C. partellus*, 3.7% for *B. fusca*, and 0.9% for *S. frugiperda.* Intraspecific divergence for individuals within *B. fusca* ranged between 0.1% and 1.9%; 0.9% and 1.6% *S. frugiperda;* 0.0 and 2.1% *C. partellus* (supp. file S1).

Haplotype analysis using DnaSP identified three different haplotypes for *S. frugiperda*, eight for *B. fusca* and three for *C. partellus*, respectively (Table 3). *S. frugiperda* COI sequence data had nine polymorphic sites (1.73%) of which eight (1.54%) were parsimony informative (Table 3). Similarly, the sequence data for *B. fusca* contained 40 segregating (7.78%) and 36 parsimony informative (7%) sites, respectively (Table 3). *C. partellus* had three polymorphic (2.09%) and two parsimony informative (1.40%) sites. Based on the sequence statistics shown in Table 3, nucleotide diversity (π) for each of the three species indicate very low genetic diversity. Haplotype distribution for all three species is shown in Table 3. All *C. partellus* specimens from Malawi were in the most common haplotype, H-3 (Table 3). There were two haplotypes (H-1 and H-2) that had *B. fusca* individuals from Malawi (Table 3).

## 4. Discussion

The cytochrome oxidase (COI) gene of the mitochondrial DNA is generally used to identify biotypes and study population genetics in insects [18,19,20,21,22]. In this study, based on phylogenetic analyses of the COI gene, larvae of Lepidopteran species infesting sugarcane in southern Malawi were identified as *Busseola fusca*, *Chilo partellus* and *Spodoptera frugiperda* (Figure 3, Figure 4 and Figure 5).

There are two cryptic species within *S. frugiperda* known as ‘species 1 or rice’ and ‘species 2 or maize or corn’ strains [35]. Both races occur in Africa [36]. The two races differ in their susceptibility to chemical and biological agents [36]. Phylogenetic analysis based on the COI gene sequence, the *S. frugiperda* sample we collected aligned with *S. frugiperda* sample from Florida in the United States of America (USA). This indicated that the *S. frugiperda* specimen was of American origin. Moreover, the *S. frugiperda* DNA sequences sample from Kaombe closely aligned *S. frugiperda* spp. 1 or ‘rice’ strains (Figure 5) from Ghana where first reports of *S. frugiperda* introduction in Africa were from [17]. DNA polymorphism analysis for this pest showed very low genetic diversity alluding to its recent introduction in Africa.

*S. frugiperda* is an invasive species that was recently introduced in Africa [16,17]. It has a strong preference for grasses [16]. Since the 2016/2017 cropping season, *S. frugiperda* has been proving to be a serious pest of maize in Malawi. So far, the Government of Malawi’s efforts on managing this pest are chiefly curative. The Food and Agricultural Organization (FAO) of the United Nations recommends the use of pheromone traps for detecting the incidence and severity of *S. frugiperda* [37]. Accurate identification of pest species is essential for effectiveness of pheromones traps as a monitoring tool [38]. Our results indicate that *S. frugiperda* infesting sugarcane in the Lower Shire Valley is the ‘rice strain.’ There is a need to ascertain if the ‘rice strain’ is the only *S. frugiperda* race infesting sugarcane in the Lower Shire Valley since both races are known to infest maize. Considering the availability of host plants throughout the year and the voracious nature of *S. frugiperda*, this species has the potential to become a serious pest of sugarcane if no effective measures are put in place to control its spread. It is also essential to determine the biology and species composition of *S. frugiperda* populations on major cereal crops of Malawi.

*B. fusca* specimens characterized in the study had 3.7% intraspecies divergence indicating the presence of geographical species [18,20,21,22]. The species had a higher haplotype diversity but low nucleotide diversity (Table 2). This indicates that there is low genetic differentiation in *B. fusca.* Our finding agrees with Assefa Y. and Dhlamini T. [18], and Peterson B.et al. [39] who reported limited sequence divergence for *B. fusca* in both Swaziland and South Africa. However, these authors did not determine genetic relatedness of their *B. fusca* insect specimens with those in other African countries. Phylogenetic analysis for *B. fusca* sequences generated in this study formed a distinct but closely related clade to *B. fusca* sequences from South Africa but was distantly related to *B. fusca* from Ethiopia and West Africa, Ghana [18,35,40]. This indicates that the *B. fusca* in southern Malawi is part of the Southern Africa population. This observation is in line with known *B. fusca* population expansion in Africa [20]. Sezonlin M. et al. [20] indicated that *B. fusca* populations in southern Africa belong to clade originate from Kenya and belong to *B. fusca* clade KII. The characteristic features for *B. fusca* clade KII are high haplotype diversity and low nucleotide diversity [20,21,22].

In this study, we have determined the identity of *Chilo* species infesting sugarcane in Southern Malawi using both morphological and the COI 1 gene barcode. It is *Chilo partellus* and not *C. sacchariphagus*. As an entire population, *C. partellus* samples sequenced in this study displayed low genetic diversity. Evidence of this is the low haplotype diversity (Hd) and nucleotide diversity (π) calculated for *C. partellus.* This agrees with previous studies done on *C. partellus* specimens from South Africa [19]. The current recommendation involving the use of the generalist egg parasitoid *T. chilonis* may be less effective. Instead, the larval parasitoid *Cotesia flavipes* commonly used in *C. partellus* classical biological control [41] should be employed.

Genetic variation within pest species may affect pest biology and the effectiveness of pest control tactics [42,43,44]. For instance, *B. fusca* morphotypes differ in their susceptibility to the main biological control agent, *Cotesia sesamiae* [20,21,41]. Similarly, genetic differentiation among *E. saccharina* populations is associated with the pest’s host preferences and its natural enemy guild in different agroecological zones of Africa [45].

This study has shown that *C. partellus* (and not *C. sacchariphagus*) *and B. fusca* are the main stemborers of sugarcane in southern Malawi. We also found that the recently invasive fall armyworm *S. frugiperda* ‘rice strain’ infested sugarcane in southern Malawi. Genetic variability was low in *B. fusca* and the majority of *C. partellus* populations. Some *C. partellus* individuals demonstrated higher genetic diversity. Accurate pest identification is the key to sustainable and effective pest control. It is important to sequence cereal stemborer species and associated natural enemies (arthropod and microbial) from all agroecological zones of Malawi in order to improve current and offer prospects for future biocontrol using microbial pesticides.

## Figures and Tables

**Table 1:** Description of reference sequences used in this study and their associated GenBank accession numbers.

Family	Genus	Species	Accession No.
Noctuidae	*Busseola*	*Fusca*	KY472246, KY472247, KM061945, KM061880, DQ337201, DQ337199
	*Spodoptera*	*frugiperda*	KY472240, KY472248, KY472250, KY472253, KY472255, GU095403 JQ547900, HM136602 HM136600, HM136599
	*Sesamia*	*inferens*	KC911715
Crambidae	*Chilo*	*partellus*	KX351380, HQ991218 KP233794, HQ990905 HQ991286, HQ991263 HQ990908, HQ991263

**Table 2:** Haplotype number and diversity in *Busseola fusca*, *Chilo partellus*, and *Spodoptera frugiperda* populations.

Species	No. of Individuals (*n*)	No. of Polymorphic Sites (S)	No. of Parsimony Informative Sites (PI)	No. of Haplotypes	Haplotype Diversity (H_d_)	Nucleotide Diversity (π)	Intraspecific Divergence (mean)
*B. fusca*	11	40	36	8	0.9273	0.036	0.037
*C. partellus*	70	3	2	3	0.220	0.003	0.003
*S. frugiperda*	11	9	8	3	0.473	0.005	0.009

**Table 3:** Distribution of *Busseola fusca*, *Chilo partellus*, and *Spodoptera frugiperda* into respective haplotypes.

Species	Haplotype	No.	Individuals
*B. fusca*	H-1 H-2 H-3 H-4 H-5 H-6 H-7 H-8	3 2 1 1 1 1 1 1	N4-1405, N64-1410, N39-1408 KO23-9206, N2-1405 KY472246 KY472247 KM061945 KM061880 DQ337201 DQ337199
*S. frugiperda*	H-1 H-2 H-3	8 1 2	S41-9005, KY472250, KY472253, GU095403, JQ547900, HM136602, HM136600, HM136599 KY472240 KY472248, KY472255
*C. partellus*	H-1	1	KX351380
	H-2	7	HQ991218, KP233794, HQ990905 HQ991286, HQ991263, HQ990908, HQ991263
	H-3	58	N2-1410, N10-1323, KO11-9206, N14-1323, N17-1405, N19-1408, KA20-6506, KA24-8324, N25-1323, KA27-8314, N28-1405, KA29-8112, N3-1410, N31-4212, KA32-8324, N33-3253, S38-9008, MB4-125, N43-3525, N44-1410, N45-1410, N46-3253, N47-1407, KA49-B540, N5-1408, KO50-9226, N51-1323, N52-3801, N53-1405, N54-2625, S56-9002, A58-6505, S59-9005, N6-1406, N60-1407, N61-1406, N62-5023, S63-9006, N65-2625, KO7-9211, S71-9006, N72-1410, N73-1323, KO74-9226, N75-5923, S76-9006, N77-2625, N78-1321, S8-9005, N80-2625, N89-1401, N91-4212, N92-5023, N96-1401, N98-1405, N99-1401, MB100, N94-1406

## Supplementary Materials

The following are available online at http://www.mdpi.com/2075-4450/9/3/74/s1, Table S1: Lepidoptera larvae sampling points in sugarcane fields located in Chikwawa and Nsanje districts, southern Malawi; supp. file S1: Sequences of representative larvae collected from sugarcane fields in Chikwawa and Nsanje District, Southern Malawi.
