# Supplementary material for: Determination of Genetic Diversity in Chilo partellus, Busseola fusca, and Spodoptera frugiperda Infesting Sugarcane in Southern Malawi Using DNA Barcodes
Source: Insects. 2018 Jun 22;9(3):74. doi: 10.3390/insects9030074 (PMC6165303; doi:10.3390/insects9030074)
Supplement: Supplementary file 1 [file insects-09-00074-s001.zip › Supplementary Table S1.pdf]

Supplementary Table 1: Lepidopteran larvae collection sites in sugarcane fields located in Chikwawa and Nsanje Districts, southern Malawi

**Location**	**Coordinates**
Alumenda	16°23'18.8"S 34°54'00.7"E
Alumenda	16°23'44.9"S 34°53'44.4"E
Alumenda	16°21'54.2"S 34°53'04.1"E
Alumenda	16°22'47.8"S 34°53'00.8"E
Alumenda	16°22'60.0"S 34°52'55.2"E
Alumenda	16°22'23.5"S 34°51'58.7"E
Kaombe	16°32'57.8"S 35°03'40.8"E
Kaombe	16°32'53.7"S 35°03'43.2"E
Kaombe	16°32'53.7"S 35°03'43.2"E
Kaombe	16°33'47.6"S 35°03'18.7"E
Kaombe	16°33'43.5"S 35°03'22.7"E
Kaombe	16°33'36.1"S 35°03'22.6"E
Kaombe	16°33'06.5"S 35°03'50.1"E
Kaombe	16°32'53.3"S 35°03'54.7"E
Kasinthula	16°07'16.9"S 34°51'37.5"E
Kasinthula	16°07'15.5"S 34°51'35.5"E
Kasinthula	16°07'12.2"S 34°51'37.0"E
Kasinthula	16°06'51.7"S 34°51'31.3"E
Kasinthula	16°06'53.5"S 34°50'55.4"E
Kasinthula	16°06'51.7"S 34°50'53.8"E
Kasinthula	16°07'45.8"S 34°49'41.5"E
Kasinthula	16°07'46.6"S 34°49'46.8"E
Kasinthula	16°08'12.4"S 34°50'07.6"E
Kasinthula	16°08'06.2"S 34°50'11.0"E
Kasinthula	16°04'54.3"S 34°47'44.8"E
Kasinthula	16°04'58.5"S 34°47'45.3"E
Kasinthula	16°05'02.7"S 34°47'30.2"E
Kasinthula	16°05'06.3"S 34°47'30.6"E
Kasinthula	16°05'17.9"S 34°47'51.2"E
Kasinthula	16°05'11.9"S 34°48'37.5"E
Kasinthula	16°05'19.6"S 34°48'41.2"E
Kasinthula	16°05'20.8"S 34°48'03.2"E
Kasinthula	16°05'21.7"S 34°46'50.9"E
Kasinthula	16°05'36.2"S 34°46'59.0"E
Kasinthula	16°05'49.9"S 34°47'20.8"E
Kasinthula	16°05'53.2"S 34°47'21.2"E
Kasinthula	16°05'48.7"S 34°46'39.2"E
Kasinthula	16°06'03.3"S 34°46'41.4"E
Kasinthula	16°06'18.1"S 34°47'37.2"E
Kasinthula	16°06'37.4"S 34°47'45.6"E
Kasinthula	16°06'38.3"S 34°47'42.6"E

Location	Coordinates
Kasinthula	16°06'40.7"S 34°48'04.0"E
Kasinthula	16°06'43.3"S 34°48'01.6"E
Kasinthula	16°06'44.3"S 34°46'42.2"E
Nchalo	16°16'47.1"S 34°54'02.1"E
Nchalo	16°14'40.9"S 34°53'42.8"E
Nchalo	16°14'41.8"S 34°53'39.7"E
Nchalo	16°14'40.9"S 34°53'42.7"E
Nchalo	16°14'45.5"S 34°53'40.0"E
Nchalo	16°14'49.7"S 34°53'32.8"E
Nchalo	16°14'46.1"S 34°53'37.4"E
Nchalo	16°15'02.2"S 34°53'37.6"E
Nchalo	16°15'02.1"S 34°53'36.5"E
Nchalo	16°15'17.0"S 34°53'50.6"E
Nchalo	16°15'16.7"S 34°53'54.8"E
Nchalo	16°15'15.9"S 34°53'54.5"E
Nchalo	16°14'49.8"S 34°54'14.5"E
Nchalo	16°14'49.5"S 34°54'15.8"E
Nchalo	16°14'48.9"S 34°54'15.4"E
Nchalo	16°14'53.7"S 34°54'08.3"E
Nchalo	16°14'52.2"S 34°54'07.0"E
Nchalo	16°15'00.5"S 34°54'10.1"E
Nchalo	16°14'59.0"S 34°54'08.2"E
Nchalo	16°14'51.0"S 34°54'24.8"E
Nchalo	16°14'54.6"S 34°54'19.8"E
Nchalo	16°14'53.9"S 34°54'20.9"E
Nchalo	16°15'03.4"S 34°54'12.9"E
Nchalo	16°14'58.7"S 34°54'23.5"E
Nchalo	16°15'01.3"S 34°54'16.9"E
Nchalo	16°15'04.6"S 34°54'13.8"E
Nchalo	16°14'56.3"S 34°54'23.1"E
Nchalo	16°14'58.7"S 34°54'16.8"E
Nchalo	16°15'20.6"S 34°54'24.1"E
Nchalo	16°15'17.6"S 34°54'27.7"E
Nchalo	16°15'14.7"S 34°54'33.8"E
Nchalo	16°15'13.8"S 34°54'36.3"E
Nchalo	16°15'41.1"S 34°54'12.3"E
Nchalo	16°15'35.8"S 34°54'00.5"E
Nchalo	16°15'28.0"S 34°53'57.6"E
Nchalo	16°15'26.3"S 34°54'01.8"E
Nchalo	16°15'26.0"S 34°54'02.6"E
Nchalo	16°15'00.2"S 34°53'22.5"E
Nchalo	16°14'57.8"S 34°53'20.0"E
Nchalo	16°14'58.4"S 34°53'16.5"E

Location	Coordinates
Nchalo	16°15'00.2"S 34°53'19.2"E
Nchalo	16°14'59.3"S 34°53'17.9"E
Nchalo	16°14'57.8"S 34°53'16.9"E
Nchalo	16°11'05.7"S 34°52'41.4"E
Nchalo	16°10'54.1"S 34°52'36.1"E
Nchalo	16°10'41.3"S 34°52'30.2"E
Nchalo	16°10'53.5"S 34°52'39.1"E
Nchalo	16°11'08.1"S 34°52'42.2"E
Nchalo	16°11'08.6"S 34°52'42.5"E
Nchalo	16°10'29.9"S 34°52'23.7"E
Nchalo	16°11'15.4"S 34°53'09.4"E
Nchalo	16°11'13.0"S 34°53'12.7"E
Nchalo	16°12'50.4"S 34°52'16.1"E
Nchalo	16°13'11.8"S 34°50'46.7"E
Nchalo	16°13'04.4"S 34°50'47.3"E
Nchalo	16°10'58.8"S 34°50'12.9"E
Nchalo	16°11'57.6"S 34°47'20.7"E
Nchalo	16°11'55.8"S 34°47'21.3"E
Nchalo	16°11'53.1"S 34°47'21.1"E
Nchalo	16°11'52.3"S 34°47'22.0"E
Sande	16°07'44.6"S 34°52'18.6"E
Sande	16°07'60.0"S 34°52'42.4"E
Sande	16°08'05.6"S 34°51'43.7"E
Sande	16°08'08.6"S 34°51'37.9"E
Sande	16°08'05.1"S 34°51'08.3"E
Sande	16°08'00.9"S 34°50'59.3"E
Sande	16°08'06.3"S 34°50'55.7"E
Sande	16°07'59.5"S 34°50'52.6"E
Sande	16°07'57.1"S 34°50'53.2"E
Sande	16°07'54.7"S 34°50'50.1"E
Sande	16°07'43.4"S 34°51'55.0"E
Sande	16°07'45.8"S 34°52'03.1"E
Mbewe	16°11'04.4"S 34°46'34.4"E
Mbewe	16°10'10.1"S 34°46'35.8"E
Mikalango	16°25'31.7"S 34°49'19.8"E
Livunzu	16°11'53.4"S 35°00'31.0"E
Livunzu	16°11'50.2"S 34°59'58.2"E
Livunzu	16°11'54.9"S 35°00'07.9"E
Kalambo	16°00'31.6"S 34°29'01.1"E
Kalambo	16°00'25.8"S 34°28'58.3"E
